# Does Platelet-Rich Plasma Treatment Increase In Vitro Fertilization (IVF) Success in the Infertile Population?

**DOI:** 10.7759/cureus.47239

**Published:** 2023-10-17

**Authors:** Sahila Safarova, Munire Funda Cevher Akdulum, Ismail Guler, Nuray Bozkurt, Ahmet Erdem, Recep O Karabacak

**Affiliations:** 1 Obstetrics and Gynaecology, Gazi University, Ankara, TUR

**Keywords:** in vitro fertilization (ivf), pregnancy, low ovarian reserve, infertility, platelet-rich plasma/ prp

## Abstract

Objectives: Platelet-rich plasma (PRP) is obtained by centrifuging the platelet-rich portion of the patient's own blood. The objective of our study is to retrospectively examine the impact of intraovarian PRP injection on infertile women with diminished ovarian reserve, specifically focusing on the oocyte count, oocyte quality, and endometrial thinning.

Methods: A retrospective assessment was conducted on cases who had intraovarian PRP injection at the in vitro fertilization (IVF) unit of the Obstetrics and Gynecology Department of Gazi University School of Medicine hospital. The review encompasses the period from 1 January 2015 to 30 June 2020. The endometrial thickness, follicle count of greater than 14 millimeters, estradiol levels, follicle-stimulating hormone (FSH) levels, and antral follicle count were assessed during the menstrual cycle both prior to and within a period of 1-6 months following the PRP procedure. Twenty nonpregnant patients who had IVF before and 4-6 months after PRP were admitted to the post-PRP IVF cycle. The quantification of oocytes and M2 oocytes was conducted both prior to and subsequent to PRP treatment.

Results: Among 120 cases, only 60 cases who fulfilled inclusion criteria were analyzed. The basal endometrial thickness, basal follicle number (>14 mm), estradiol value, oocyte count, and M2 oocyte count exhibited a statistically significant increase following the administration of PRP injection (p<0.001). The basal FSH value exhibited a notable drop following the administration of PRP injection, with a statistically significant difference (p=0.002). In the pregnant group, the number of oocytes obtained with oocyte pick-up (OPU) increased by 300%, and in the nonpregnant group, the increase was 125% only. The number of M2 oocytes obtained with OPU increased by 250% in the pregnant group, while it was 93% in the nonpregnant group.

Conclusion: Ultimately, the affordability of PRP production and its considerable theoretical efficacy have the potential to substantially decrease the expenses associated with assisted reproductive technology procedures. In the present scenario, the administration of an intraovarian PRP injection may be contemplated as a therapeutic intervention for women exhibiting diminished ovarian reserve.

## Introduction

Platelet-rich plasma (PRP) is the platelet-rich plasma fraction in the upper part of autologous blood, and it is a new treatment option in modern medicine known as 'orthobiological'. PRP is obtained by centrifuging the platelet-rich portion of the patient's own blood. This material is injected into the damaged tissue. Platelet alpha-granules are enriched in growth factors that enhance tissue repair, including platelet-derived growth factor, insulin-like growth factor, transforming growth factor-beta, and vascular endothelial growth factor. The main objective of the procedure is to stimulate tissue repair by delivering autologous platelets at supraphysiological concentrations to the damaged tissue [1,2].

With changes in social and behavioral life in the modern era, pregnancy and delivery have tended to be postponed to later ages. Fertility issues arise as maternal age rises. Although it is hypothesized that intraovarian injections of PRP increase pregnancy rates in patients with lower ovarian reserve, a paucity of studies in the literature limits the inappropriate use of PRP application in infertile women [3,4].

In several case series and studies on intraovarian PRP injection, spontaneous or in vitro fertilization (IVF) pregnancies in women with primary ovarian insufficiency (POI) after injections have been reported [5-8]. There are studies that suggest that intraovarian PRP injection dramatically enhances the number of oocytes, M2 oocytes, and grade 1 embryos in women with limited ovarian reserve [5,9,10]. Although the mechanism of action is unknown, it has been proposed that these findings are due to platelet-derived cytokines, which may influence de novo oocyte growth, and that PRP enhances the ovarian microenvironment and boosts ovarian vascular activity [6-8].

The aim of our study is to retrospectively reveal the effects of intraovarian PRP injection applied to infertile women with low ovarian reserve on oocyte count, oocyte quality, and thin endometrium. In addition, we aimed to investigate the pregnancy rates that may occur with spontaneous and assisted reproductive technologies in these women.

## Materials and methods

The cases who underwent intraovarian PRP injection in the IVF unit of the Obstetrics and Gynecology Department of Gazi University School of Medicine hospital between 1 January 2015 and 30 June 2020 were retrospectively reviewed. The study has received approval from the local ethics committee (2020-417). Informed consent was waived because the study was retrospective.

Parameters such as patient age, duration and cause of infertility, number of previous IVF attempts, PRP application number, and spontaneous and IVF pregnancies after PRP application were evaluated. 

Poor ovarian responders (PORs) were defined as women having at least two of the following three criteria: (i) advanced maternal age ≥ 40 or any other risk factor for the poor ovarian response; (ii) a previous poor ovarian response (cycles cancelled or ≤ 3 oocytes retrieved with a conventional protocol); (iii) an abnormal ovarian reserve test (antral follicle count (AFC) 4 or less follicles or anti-Müllerian hormone (AMH) < 0.5 - 1.1 ng/ml) according to European Society of Human Reproduction and Embryology (ESHRE) Bologna criteria [11]. 

We included infertile (who could not become pregnant for one year) patients with low ovarian reserve 20 -50 years of age. Patients with a history of cancer, major lower abdominal surgery, male infertility, and anticoagulant therapy were excluded from the study. Among 120 cases, only 60 cases who fulfilled inclusion criteria were analyzed. 

Endometrial thickness, follicle count of >14mm, estradiol (patients with estradiol values up to 40 pg/ml were included.), follicle-stimulating hormone (FSH), and AFC measurements were measured during the menstrual period before and 1-6 months after the PRP procedure. All patients who did not get spontaneously pregnant were recruited for IVF after three months.

Twenty nonpregnant patients who had IVF before and 4-6 months after PRP were admitted for the post-PRP IVF cycle. The numbers of oocytes and M2 oocytes retrieved were measured before and after PRP. 

The AMH value could only be measured in 10 patients due to insurance reimbursement problems. Because AMH is not affected by the menstrual cycle, it can be measured on any day of the cycle.

Venous blood 8.5 ml is drawn from the antecubital region and placed in a 10 ml syringe with 1 ml of sterile citrate (3% citrate-phosphate-dextrose (CPD)). After transferring the citrated blood into a 15 ml flat-bottom sterile tube, the mixture is centrifuged for 5 minutes at 2100 revolutions per minute (857 g) (Heraeus Labofuge 400 Thermo Scientific Germany). Following centrifugation, the 'buffy-coat' fraction, which is between the erythrocyte fraction at the bottom of the tube and the upper fraction, is drawn into the 5 ml syringe containing 0.1 ml calcium gluconate (for platelet activation) through the attached 16 G needle in 0.6ml volume. All processes were carried out at room temperature, and PRP injections with an intraovarian total volume of 0.6 ml were performed under aseptic conditions using a 17-G (Ovum Pick-Up) needle in 10 minutes.

Continuous variables were represented by mean, standard deviation, and/or median (minimum-maximum), whereas categorical data were represented by numbers and percentages. ((Next (new value) - Previous (basal) value) / previous (basal) value) *100) was used to calculate the percent change. The Kolmogorov-Smirnov Goodness of Fit Test was used to analyze the normality of continuous variables. When the data did not fit a normal distribution, the Kruskal-Wallis test was used to compare the three groups, and the Mann-Whitney U test was used to compare the two groups. In dependent groups, intragroup comparisons were made using the T-Test. To compare categorical data, the Chi-square test was used. IBM SPSS Statistics for Windows, Version 26 (Released 2019; IBM Corp., Armonk, New York, United States) was used for the analyses. Cases with a type 1 error level of less than 5% were considered statistically significant.

## Results

The average age of the 60 infertile women undergoing intraovarian PRP was 36.5 ±0.8 years. The mean BMI of the patients was found to be 26.2±0.4 kg/m^2^, the mean duration of marriage was 5.2±3.3 years, and the duration of infertility was 3.8±2.6 years (Table 1).

**Table 1:** The socio-demographic (age, BMI, duration of marriage, duration and cause of infertility, previous IUI-IVF, and abortion history) and clinical (medical examinations (PRP application session)) characteristics of the patients. BMI: Body mass index, IUI: intrauterine insemination, IVF: in vitro fertilization, PRP: platelet-rich plasma.

		N=60
Age (year) (Mean ± SD)		36.5 ± 0.8
BMI (kg/m^2^) (Mean ± SD)		26.2 ± 0.4
Marriage duration (year) (Mean ± SD)		5.2±3.3
Infertility duration (year) (Mean ± SD)		3.8±2.6
Cause of infertility (N, %)	Primary	43 (%71.7)
	Secondary	17 (%28.3)
IUI count (N, %)	0	44 (%73,3)
	1	4 (%6,7)
	2	6 (%10)
	≥3	6 (%10)
IVF count (N, %)	0	28 (%46.7)
	1	10 (%16.7)
	2	11 (%18.3)
	≥3	11 (%18.3)
Abortion count (N, %)	0	51 (%85)
	1	7 (%11.7)
	≥2	2 (%3.3)
PRP application session (N, %)	1 session	52 (%86.7)
	2 sessions	5 (%8.3)
	3 sessions	3 (%5)

While 32 (53.3%) of the participants had at least one history of IVF, 16 (26.7%) of the participants had at least one history of intrauterine insemination (IUI), and 9 (15%) had a history of abortion. Forty-three (71.79%) of the participants were primary infertile women, and 17 (28.1%) were secondary infertile women. Among secondary infertile patients, eight had live births (47%), seven had one abortion (41.2%), and two had two or more abortions (11.8%).

One session of PRP was administered to 52 patients (86.7%), two sessions to five patients (8.3%), and three sessions to three patients (5%).

Table 2 shows the laboratory values and endometrial thickness values measured in the first seven days of menstruation before and after intraovarian PRP application. The basal endometrial thickness, basal follicle number (>14 mm), estradiol value, oocyte count, and M2 oocyte count all increased significantly after PRP injection (p<0.001). The basal FSH value decreased significantly after PRP injection (p=0.002). Before and after PRP injection, there were no significant differences in AFC or AMH levels (p>0.005).

**Table 2:** Increase in the endometrial thickness, follicle count >14 mm, E2 level, number of oocytes retrieved, number of M2 oocytes, FSH level, AMH level, number of antral follicle count, and mean values before and after intraovarian PRP application. *The paired samples t-test E2: Estradiol, FSH: follicle-stimulating hormone, AMH: anti-Mullerian hormone, AFC: antral follicle count, PRP: platelet-rich plasma

	Before PRP (Mean±SD) (N=60)	After PRP (Mean±SD) (N=60)		P*
Endometrial thickness (mm)	4.4 ± 0.2	9.6 ± 0.3	<0.001	
Follicle count (>14 mm)	0.7 ± 0.1	3.1 ± 0.2	<0.001	
E2 (pg/mL)	86.2 ± 11.3	466.1 ± 54.3	<0.001	
Oocyte count (N=20)	0.4 ± 0.2	2.21 ± 0.3	<0.001	
M2 oocyte count (N=20)	0.18 ± 0.14	1.44 ± 0.26	<0.001	
FSH (IU/L)	12.9 ± 1.1	11.1 ± 1.1	0.002	
AMH (N=10) (ng/mL)	0.8 ± 0.3	2.7 ± 0.8	NS	
AFC	4.6 ± 0.4	4.9 ± 0.3	NS	

Laboratory parameters and endometrial thickness were compared before and six months after PRP application based on pregnancy status (Table 3). In the pregnant group, the number of oocytes obtained with oocyte pick-up (OPU) increased by 300%, in the nonpregnant group, the increase was 125% only. The number of M2 oocytes obtained with OPU increased by 250% in the pregnant group, and 93% in the nonpregnant group. The pregnant group had a significant increase in the number of oocytes and M2 oocytes. While the number of antral follicles decreased by 7% in the spontaneous pregnancy group, AFC increased by 43% and 37% in the nonpregnant and assisted reproductive technology (ART) pregnancy groups, respectively. Although these increases are not significant, they are in trend (p=0.06). It supports the notion that follicle quality is more important than follicle quantity.

**Table 3:** Comparison of endometrial thickness increase, >14 mm follicle count, E2 level, number of oocytes retrieved, M2 oocyte count, and FSH level before and after PRP application according to the pregnancy status. * Kruskal Wallis Test (Mann-Whitney U Test for pairwise comparisons). ART: Assisted reproductive technology, PRP: platelet-rich plasma, E2: estradiol, FSH: follicle-stimulating hormone, AFC: antral follicle count

	Pregnancy (N=17)						No Pregnancy (N=43)			P*
	Spontaneous (N=13)			Assisted Reproductive Technology (ART) (N=4)						
	Mean change (Mean±SD)		Percent change(%)	Mean change (Mean±SD)		Percent change (%)	Mean change (Mean±SD)		Percent change (%)	
	Before PRP	After PRP		Before PRP	After PRP		Before PRP	After PRP		
Endometrial thickness increase (Mean±SD)	4.5 ± 2	10 ± 2	126	4.4 ± 2	10 ± 1	127	4.1 ± 1	9.2 ± 3	120	NS
>14 mm follicle count (Mean±SD)	1 ± 1	3.5 ± 1	250	1.5 ± 1	4.5 ± 1	213	0.92 ± 1	2.8 ± 2	204	NS
Estradiol level (E2) (pg/mL) (Mean±SD)	53 ± 25	459 ± 404	766	86 ± 31	357 ± 247	315	59 ± 97	484 ± 420	720	NS
follicle stimulating hormone (FSH) level (IU/L) (Mean±SD)	9.4 ± 5	8 ± 3	-14.8	8 ± 4.4	6.7 ± 3	-16.2	13.7 ± 10	12 ± 9	-12.4	NS
Oocyte count (N=20) (Mean±SD)				0.75 ± 0	3 ± 1.8	300	0.75 ± 1.4	1.7 ± 1.8	125	0.004
M2 oocyte count (N=20) (Mean±SD)				0.7 ± 0	2.5 ± 1.3	250	0.5 ± 0.2	0.98 ± 1.6	93	0.002
Antral follicle count (AFC) (Ort±SE)	5.7 ± 4.3	5.3 ± 2	-7	3.5 ± 3.3	4.9 ± 2.3	36.9	3.2 ± 2.6	4.6 ± 2.5	43	0.064

There was a spontaneous pregnancy rate in patients with three years or less infertility compared to those with more than three years of infertility which was 31.4 -8 % respectively, significantly favoring for less infertility duration. There was no pregnancy less than three years of infertility cases in the IVF group which meant that the short duration of infertility cases convinced them to wait after PRP injection, with longer than three years preferred to admit to IVF immediately in four cases (p=0.009). Our study shows that as the duration of infertility increases, spontaneous pregnancy rates will decrease in patients undergoing PRP, but they may become pregnant through IVF (Table 4).

**Table 4:** Comparison of PRP application with medical examinations (HSG, PRP application session) and socio-demographic characteristics (age, BMI, duration of marriage, duration and cause of infertility, and IUI, IVF and abortion history) according to the pregnancy status. *Chi-square test ART: Assisted reproductive technology, HSG: hysterosalpingography, PRP: platelet-rich plasma, BMI: body mass index, IUI: intrauterine insemination, IVF: in vitro fertilization.

		Pregnancy + (N=17)		No Pregnancy (N=43)	P*
		Spontaneous (N=13)	ART (N=4)		
HSG (N, %)	Normal	12 (24)	3 (6)	35 (70)	NS
	Pathological	1 (10)	1 (10)	8 (80)	
PRP application session (N, %)	1. session	10 (19.2)	4 (7.7)	38 (73.1)	NS
	2. sessions	3 (60)	0(0)	2 (40)	
	3 sessions	0 (0)	0 (0)	3 (100)	
Age(year) (N, %)	<35	6 (27.3)	2 (9.1)	14 (63.6)	NS
	≥35	7 (18.4)	2 (5.3)	29 (76.3)	
BMI(kg/m^2^) (N, %)	<25	3 (15)	0 (0)	17 (85)	NS
	25-29	6 (20.7)	4 (13.8)	19 (65.5)	
	≥30	4 (40)	0 (0)	6 (60)	
Duration of marriage (year) (N, %)	≤4	9 (29)	1 (3.2)	21 (67.7)	NS
	>4	4 (13.8)	3 (10.3)	22 (75.9)	
Duration of infertility (N, %)	≤3	11 (31.4)	0 (0)	24 (68.6)	0.009*
	>3	2 (8)	4 (16)	19 (76.0)	
Cause of infertility (N, %)	Primary	12 (27.9)	3 (7)	28 (65.1)	NS
	Secondary	1 (5.9)	1 (5.9)	15 (88.2)	
IUI history (N, %)	Yes	4 (25)	1 (6.3)	11 (68.8)	NS
	No	9 (20.5)	3 (6.8)	32 (72.7)	
IVF history (N, %)	Yes	10 (31.2)	0 (0)	22 (68.75)	NS
	No	3 (10.7)	4 (14.28)	21 (75)	
Abortion history (N, %)	Yes	2 (22.2)	0 (0)	7 (77.8)	NS
	No	11 (21.6)	4 (7.8)	36 (70.6)	

Due to the retrospective nature of our study, we could not reach all pregnant cases' birth data and the live birth rate could not be calculated.

## Discussion

Infertility is an important issue for the survival of humanity. Ovarian tissue ages faster than other tissues in the body, as the woman gets older. A decrease in the number of follicles leads to a decrease in oocyte quality and a gradual decrease in women's pregnancy and live birth rates. After the age of 35, monthly fertility in fertile women decreases from 20% to 10%, and the risk of infertility increases in women with low ovarian reserve. The mean age of the patients in our study was 36 years which seems to be consistent with other studies [12,13].

In our study, low ovarian reserve infertile patients had a pregnancy rate of 28.4%. Among these patients, a subgroup who has less than three years of infertility was significantly more likely to spontaneously conceive after PRP application (32.5% pregnancy rate). 

Gjönnaess et al. reported that 51 (86%) of 62 patients with polycystic ovary syndrome had regular cycles after ovary drilling [14]. Gjønnaess discovered that ovulation rates differed depending on the number of holes he opened on the surface of the ovary in this study. According to Gjønnaess, ovulation occurs at 66.6% when less than six holes are made on the ovary surface, 92% when 6-10 holes are opened, and 96% when more than 10 holes are opened [15]. In our study, there was no spontaneous pregnancy after needle insertions during OPU in 32 patients (53.3% of patients), who had previously undergone IVF before PRP application, whereas spontaneous pregnancy was observed in 10 (31.2%) of 32 patients who had previously undergone IVF after PRP application. As a result, the absence of pregnancy in patients, who had previously undergone OPU (despite needle access), and the occurrence of pregnancy following PRP application suggest that this pregnancy rate is due to the physiological effect of PRP itself.

The number of spontaneous pregnancies increased as the number of PRP applications increased. Ten of the 52 patients who received one session of PRP experienced spontaneous pregnancy (19.1% pregnancy rate). Three of five patients who received two sessions of PRP experienced spontaneous pregnancy (60%). This situation is insignificant (p=0.12 in the chi-square / Fisher Test). However, it is unclear whether the increased pregnancy rate is due to the increased number of PRP in the PRP sessions or the increased number of needles entering the ovary, it may be a combination of both. Since there was no control group in this retrospective study, the pregnancy rates of the patients with needle insertion only and left without treatment could not be compared, which is the weak part of our study. However, patients with poor ovarian reserve generally do not give consent, when it is recommended to wait without receiving any treatment, and even change institutions for immediate treatment. Our study group with an infertility duration of three years or less agreed to wait 1-6 months until ART treatment after PRP, and 1/3 of them became pregnant. Patients with poor ovarian reserve to wait six months after PRP application with a reasonable spontaneous pregnancy rate, according to the result of our study, as a convincing option if the infertility period is three years or less.

A comparative study by Çakiroglu et al. examined the impact of intraovarian PRP application on 510 women with poor ovarian reserve. After PRP application, 22 women (4.3%) spontaneously became pregnant, 14 women (2.7%) were excluded from follow-up, and 474 women (92.9%) received ART and showed increased AMH and AFC. Post-PRP-ART patients had 20.5% pregnancy, 12.9% live birth rate, and spontaneous pregnancy between three and six months. No significant decrease in serum FSH was observed (p=0.87) [16]. In our study, PRP was applied to 60 patients with low ovarian reserve; there were no significant differences in serum AMH levels or AFC, but there were a significant increase in serum estradiol and a significant decrease in serum FSH. We had more pregnancies after PRP rather than IVF treatment itself, only one patient with previous IVF got pregnant one month after PRP application, and 16 pregnancies occurred in 3-6 months after PRP application. Of the 28.4% clinical pregnancy rate, 21.7% (13 patients) were pregnant spontaneously and 6.7% (4 patients) were pregnant with ART. In our study, non-PRP applied followed by the same period control group is missing.

After PRP injection, AMH, FSH, and LH levels were higher in 17 women with a poor ovarian response and seven women with POI, according to Aflatoonian et al. E2 levels did not change after six weeks, but FSH levels decreased and E2 and AMH levels increased insignificantly after one month. FSH and AMH fall lasted two months. After PRP application, eight (47%) PORs became pregnant spontaneously [17]. This study found a 100% increase in serum estradiol in the first seven days of the cycle and a 14% decrease in serum FSH in the one to six months after PRP application.

In a systematic review study by Panda et al., a significant increase was observed in the serum AMH level and AFC after PRP application compared to before, while serum FSH was observed. They also reported that there was a decrease in the level of quality, but also a significant increase in the number of quality oocytes and embryos [18]. Similarly, no statistically significant changes in serum AMH and AFC were observed in our study, and a 14% decrease in serum FSH levels was observed. Concurrently, there was a notable increase in the quantity of oocytes and M2 oocytes acquired from a cohort of 20 patients (constituting 33.3% of the overall patient population) who exhibited infertility prior to the administration of PRP and subsequently underwent IVF within six months. Specifically, the number of oocytes observed a fourfold increase, while the number of M2 oocytes exhibited a sevenfold increase when compared to the before PRP application period.

The application of PRP has opened a new horizon in many fields of medicine, especially in reproductive medicine and infertility treatment [19]. Although there is a need for controlled randomized studies on the application of PRP, it seems difficult to study in terms of homogenization of patient groups. Because of the advanced age and infertility of the patients, who underwent intraovarian PRP, forming a control group that did not receive any treatment seems to be incompatible in terms of medical ethics and patient morale. As a result, we were unable to define the control group from our retrospective patient population.

Although our study yielded positive results, retrospective design is a limitation. There is a need for randomized controlled trials comparing the outcomes of intraovarian PRP injection to a sufficient number of patients. Another limitation is the lack of longer-term follow-up findings. It is unknown what the effects and persistence of repeated doses of tissue healing are. Long-term studies on when the effect disappears after six months are also needed. Long-term outcomes of applications with multiple sessions can be examined. On the other hand, no study in the literature answers questions about the quality of the applied PRP, because there is no agreement on the method of preparation of the PRP preparation and the platelet concentration in the PRP is affected by conditions such as the patient's age and hematological and chronic diseases.

## Conclusions

Eventually, the use of PRP therapy at a reduced cost has demonstrated noteworthy efficacy in achieving pregnancy rates within a timeframe of less than three years of infertility. This comparison to durations exceeding three years may yield a substantial decrease in costs associated with ART treatments for individuals with low ovarian reserve. An intraovarian PRP injection can be considered as a therapy in women with low ovarian reserve and infertility for less than three years. Its clinical efficacy must be demonstrated in randomized controlled trials. To understand the factor responsible for increased pregnancy as a result of low ovarian reserve and infertility lasting less than three years, patients who wait only up to six months and do not receive treatment, patients who only undergo ovarian puncture, and patients who receive PRP injection into the ovaries themselves should be compared.
